# Global and country-level estimates of human population at high altitude

**DOI:** 10.1073/pnas.2102463118

**Published:** 2021-04-26

**Authors:** Joshua C. Tremblay, Philip N. Ainslie

**Affiliations:** ^a^Centre for Heart, Lung and Vascular Health, University of British Columbia Okanagan, Kelowna, BC V1V 1V7, Canada

**Keywords:** global population distribution, hypoxia, mountain, geographic information system, global health

## Abstract

Estimates of the global population of humans living at high altitude vary widely, and such data at the country level are unavailable. Herein, we use a geographic information system (GIS)-based approach to quantify human population at 500-m elevation intervals for each country. Based on georeferenced data for population (LandScan Global 2019) and elevation (Global Multiresolution Terrain Elevation Data), 500.3 million humans live at ≥1,500 m, 81.6 million at ≥2,500 m, and 14.4 million at ≥3,500 m. Ethiopia has the largest absolute population at ≥1,500 m and ≥2,500 m, while China has the greatest at ≥3,500 m. Lesotho has the greatest percentage of its population above 1,500 m, while Bolivia has the greatest at ≥2,500 m and ≥3,500 m. High altitude presents a myriad of environmental stresses that provoke physiological responses and adaptation, and consequently impact disease prevalence and severity. While the majority of high-altitude physiology research is based upon lowlanders from western, educated, industrialized, rich, and democratic countries ascending to high altitude, the global population distribution of high-altitude residents encourages an increased emphasis on understanding high-altitude physiology, adaptation, epidemiology, and public health in the ∼500 million permanent high-altitude residents.

High altitude introduces environmental stressors that are distinct from sea level. Hypobaric hypoxia, colder and drier climates, and increased radiation challenge reproductive success (1). In addition to the direct effects of high altitude, the health of high-altitude residents (particularly in rural communities) is compounded by vulnerability to food and water insecurity associated with land degradation and the concurrence of conflict and climate change (2, 3). Population estimates for the number of humans residing at high altitude varies widely. Older estimates were either guesses or based upon assigning a percentage of select countries’ populations residing at high altitude (4–6). Cohen and Small (7) produced gridded population estimates based on censuses and coregistered these demographic data with elevation data, estimating that 389.4 million live at >1,500 m. The prevailing assumption is that over 140 million humans live above 2,500 m. This is based on the 139 million estimated to be residing above 2,500 m in 1995 which was calculated by estimating the percentage of populations residing above 2,500 m in select countries (table 1 in ref. 8). However, studies using geographic information system (GIS)-based approaches with updated georeferenced data (9) have estimated that, in 2010, there were 83 million residents at >2,500 m (10), and, in 2017, 74.9 million lived at >2,500 m (2). These reports do not provide country-level estimates and provide population estimates only for select elevation ranges/thresholds. To understand the impact of life at high altitude on human physiology, adaptation, health, and disease, it is imperative to know how many humans live at high altitude and where they live. Herein, we estimate that 81.6 million humans live above 2500 m, and we provide population estimates at 500-m elevation intervals for every country.

## Results

Over 500 million humans live at ≥1,500 m (6.58% of the total population), 219 million at ≥2,000 m (2.88% of the total population), 81.6 million at ≥2,500 m (1.07% of the total population), 25.2 million at ≥3,000 m (0.33% of the total population), 14.4 million at ≥3,500 m (0.19% of the total population), 6.4 million at ≥4,000 m (0.084% of the total population), 2 million at ≥4,500 m (0.027% of the total population), and 0.31 million at ≥5,000 m (0.004% of the total population). Table 1 presents the standard and model atmosphere-calculated barometric pressure, partial pressure of inspired oxygen, and global populations at 500-m elevation intervals.

**Table 1.** Barometric pressure calculated using standard and model atmospheres equations, the inspired partial pressure of oxygen (P_I_O_2_; absolute and percentage of sea level), and global population estimates at 500-m elevation intervals

	Standard atmosphere*						Model atmosphere^†^					
Altitude, m	Barometric pressure 5 °C (mmHg)	Barometric pressure 20 °C (mmHg)	P_I_O_2_ 5 °C (mmHg)	P_I_O_2_ 20 °C (mmHg)	P_I_O_2_ 5 °C (% of sea level)	P_I_O_2_ 20 °C (% of sea level)	Barometric pressure (mmHg)	P_I_O_2_ (mmHg)	P_I_O_2_ (% of sea level)	Population (millions)	% Population	
<500	>715	>717	>140	>140	>94	>94	>718	>140	>94	5 821.54	76.555	
500 to 999	671 to 715	676 to 717	131 to 140	132 to 140	88 to 94	88 to 94	679 to 718	132 to 140	89 to 94	839.582	11.041	
1,000 to 1,499	630 to 671	636 to 676	122 to 131	123 to 132	82 to 88	83 to 88	641 to 679	124 to 132	83 to 89	442.937	5.825	
1,500 to 1,999	591 to 630	599 to 636	114 to 122	116 to 123	76 to 82	77 to 83	605 to 641	117 to 124	78 to 83	281.185	3.698	
2,000 to 2,499	554 to 591	563 to 599	106 to 114	108 to 115	71 to 76	72 to 77	570 to 604	109 to 117	73 to 78	137.585	1.809	
2,500 to 2,999	519 to 554	529 to 563	99 to 106	101 to 108	66 to 71	68 to 72	537 to 570	103 to 109	69 to 73	56.352	0.741	
3,000 to 3,499	485 to 519	497 to 529	92 to 99	94 to 101	61 to 66	63 to 68	505 to 537	96 to 103	64 to 69	10.786	0.142	
3,500 to 3,999	454 to 485	467 to 497	85 to 92	88 to 94	57 to 61	59 to 63	475 to 505	90 to 96	60 to 64	8.015	0.105	
4,000 to 4,499	424 to 454	437 to 467	79 to 85	82 to 88	53 to 57	55 to 59	447 to 475	84 to 90	56 to 60	4.385	0.058	
4,500 to 4,999	396 to 424	410 to 437	73 to 79	76 to 82	49 to 53	51 to 55	420 to 447	78 to 84	52 to 56	1.711	0.023	
≥5,000	≤396	≤410	≤73	≤76	≤49	≤51	≤420	≤78	≤52	0.313	0.004	

Barometric pressure is also impacted by latitude (higher at the equator) and season. P_I_O_2_ = (barometric pressure – 47 mmHg) × 0.2093.

* Barometric pressure = 760 × [1 − 0.0065 × elevation in meters/(273.15 + temperature)]^5.255^.

^†^ Barometric pressure = exp(6.63268 − 0.1112 × elevation in kilometers − 0.00149 × elevation in square kilometers).

**Fig. 1.**
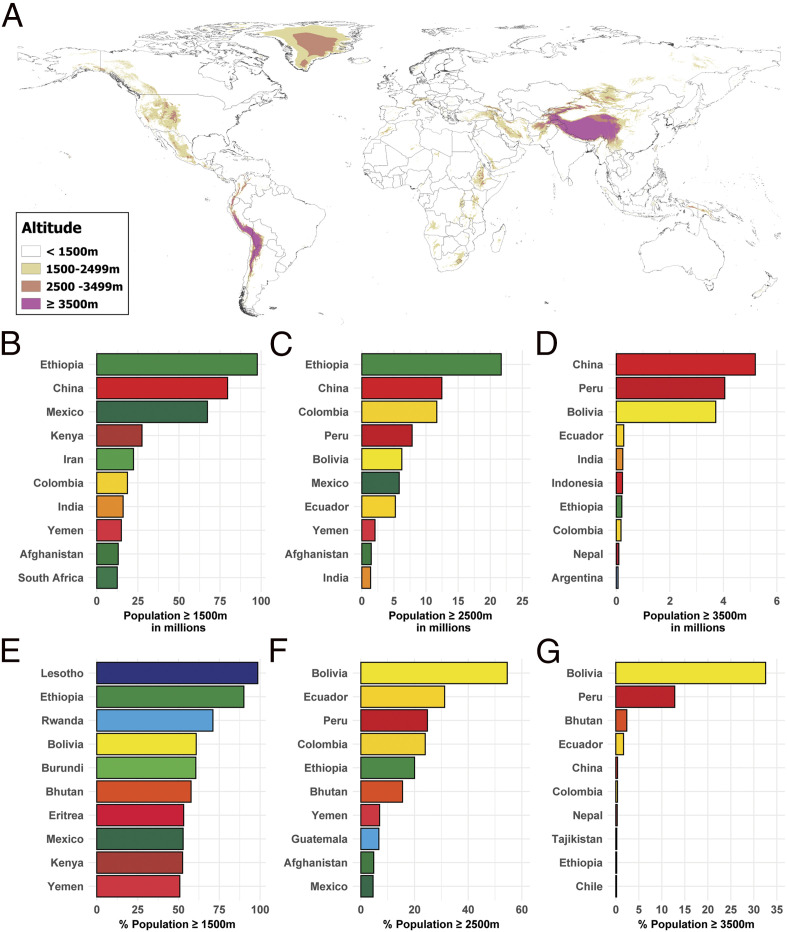
(*A*) Global map illustrating areas with altitudes of 1,500 m to 2,500 m, 2,500 m to 3,500 m, and ≥3,500 m (Antarctica is excluded). The 10 countries with the greatest total (*B*–*D*) and relative (*E*–*G*) populations at ≥1,500 m, ≥2,500 m, and ≥3,500 m are presented.

Fig. 1 illustrates that high-altitude regions are present in all continents and shows the 10 most populated countries with altitudes of ≥1,500 m, ≥2,500 m, and ≥3,500 m, and the 10 countries with the greatest percentage of national population residing at ≥1,500 m, ≥2,500 m, and ≥3,500 m. Dataset S1 contains the estimated population at 500-m elevation intervals for every country.

## Discussion

We used georeferenced population and elevation data to estimate the global and country-level population of humans at high altitude. We estimate that 81.6 million humans live at ≥2,500 m, and that these humans are primarily from non-Western countries. This figure is considerably less than non-GIS−based estimates (8) and expands upon GIS-based estimates (2, 9, 10) by providing country-level estimates at 500-m elevation intervals. We present population for 500-m intervals to provide flexible interpretation of “high altitude,” as the threshold for high altitude to elicit a physiological response varies between individuals and populations. Research is encouraged in countries with considerable high-altitude populations, to understand how the environmental stress (physiological and social) of high altitude impacts physiology, adaptation, health, and disease.

### Where Do Humans Live at High Altitude?

Humans reside at high altitude in each continent; notably, the majority of humans at high altitude live in non-Western countries. As with medical research generally (11), high-altitude research has focused on the lowlander of European descent ascending to high altitude (12). Select permanent high-altitude populations have been studied, with considerable differences in physiological measures reported (1); however, the populations studied represent just a fraction of humans at high altitude. Among the studied populations, differences in candidate genetic variants that may be adaptive and their phenotypic associations have been identified (1, 10). The distribution of where humans live at high altitude, presented herein, and their duration of high-altitude residence affords a broad natural experiment to better define the relationships between genotype and phenotype and provide insight into the evolutionary and mechanistic bases of physiological adaptations to high altitude. Further, an expanded understanding of the potential physiological differences arising from life at high altitude will lead to improvements in the diagnosis and treatment of disease, ultimately improving the health and well-being of high-altitude populations.

### Limitations.

Mobile, nomadic, or pastoralist populations and those with lowest income and most at risk are underrepresented in census data (13). Therefore, we have likely underestimated the most vulnerable populations and may have disproportionately undercounted rural high-altitude populations. The LandScan Global datasets are not recommended to be used as a change or migration tool, as the LandScan database is constantly improving its input data (14). Further, the LandScan dataset provides only a population count and not demographics (e.g., age, sex, ethnicity, etc.). Nevertheless, LandScan is the community standard for global population distribution.

## Materials and Methods

Population estimates were calculated using QGIS 3.4.0-Madeira software. Population data were acquired from Oak Ridge National Laboratory’s LandScan Global 2019 dataset (14). LandScan provides an ambient population count for cells with a spatial resolution of 30 arc-seconds (∼1 km^2^). LandScan uses the best available census counts to estimate population and applies a spatial distribution model that includes land cover, roads, slope, urban areas, village locations, and high-resolution imagery analysis. The mean elevation data from the Global Multiresolution Terrain Elevation Data (GMTED2010) were also acquired at a resolution of 30 arc-seconds (∼1 km^2^) (15). Country borders were acquired from the Database of Global Administrative Areas (GADM, version 3.6; https://gadm.org/download_country_v3.html). Further details on the methodology are provided in *SI Appendix*.

## Supplementary Material

Supplementary File

Supplementary File

## Data Availability

All study data are included in the article and Dataset S1.

## Change History

May 3, 2021: Fig. 1 and Dataset S1 have been updated.
