# Supplementary material for: Global and country-level estimates of human population at high altitude
Source: Proc Natl Acad Sci U S A. 2021 Apr 26;118(18):e2102463118. doi: 10.1073/pnas.2102463118 (PMC8106311; doi:10.1073/pnas.2102463118)
Supplement: Supplementary File [file pnas.2102463118.sapp.pdf]

Materials and Methods

To include low-altitude population estimates extending beyond cartographic maritime boundaries, countries with a maritime border were buffered 0.00833333 decimal degrees (approximately 1 km) with one segment and round end cap and join style. The difference algorithm was subsequently used for each neighboring country to prevent border overlapping; only the maritime border was extended ~1 km. Each maritime border was then visually inspected to ensure all cells containing a population value were included in the study; polygon features were drawn to encompass any population cells that fell outside of the buffered country border. For landlocked countries and maritime countries following the maritime border buffering, a point grid with horizontal and vertical spacing of 0.00833333 decimal degrees (one point in each approximately 1 km^2) was created using the country layer extent. This grid was clipped based on the country layer, so that only overlapping points remained. Population and elevation raster values (from the LandScan (1) and Global Multi-resolution Terrain Elevation Data [GMTED2010] (2) layers) were sampled from the clipped point grid layer (each cell contained one point that was sampled). These data were exported as comma separated value (.csv) files and imported into R version 3.6.1 where the dataset was filtered by 500m elevation intervals (< 500m, 500-999m, 1000-1499m, 1500-1999m, 2000-2499m, 2500-2999m, 3000-3499m, 3500-3999m, 4000-4499m, 4500-4999m and ≥ 5000m) and the sum of the population calculated for each elevation interval. This was repeated for each country and the sum of all countries used as the global population estimates.

References

1. Rose AN, *et al.* (2020) LandScan 2019. (Oak Ridge National Laboratory, Oak Ridge, TN).
2. Danielson JJ & Gesch DB (2011) *Global multi-resolution terrain elevation data 2010 (GMTED2010)* (US Department of the Interior, US Geological Survey).
